# Presence of the tunicate *Asterocarpa humilis* on ship hulls and aquaculture facilities in the coast of the Biobío Region, south central Chile

**DOI:** 10.7717/peerj.3672

**Published:** 2017-08-14

**Authors:** Javier Pinochet, Jean-Charles Leclerc, Antonio Brante, Claire Daguin-Thiébaut, Christian Díaz, Florence Tellier, Frédérique Viard

**Affiliations:** 1 Departamento de Ecología, Facultad de Ciencias, Universidad Católica de la Santísima Concepción, Concepción, Chile; 2 Centro de Investigación en Biodiversidad y Ambientes Sustentables (CIBAS), Universidad Católica de la Santísima Concepción, Concepción, Chile; 3 Magíster en Ecología Marina, Facultad de Ciencias, Universidad Católica de la Santísima Concepción, Concepción, Chile; 4 UMR 7144, Laboratoire “Adaptation et Diversité en Milieu Marin”, Team Div&Co, Station Biologique de Roscoff, Sorbonne Universités, Université Pierre et Marie Curie (Paris VI), CNRS, Roscoff, France; 5 Departamento de Medio Ambiente y Energía, Facultad de Ingeniería, Universidad Católica de la Santísima Concepción, Concepción, Chile

**Keywords:** Ascidians, DNA barcoding, Non-native species, Maritime trade, Vectors

## Abstract

Non-native ascidians are important members of the fouling community associated with artificial substrata and man-made structures. Being efficient fouling species, they are easily spread by human-mediated transports (e.g., with aquaculture trade and maritime transports). This is exemplified by the ascidian *Asterocarpa humilis* which displays a wide distribution in the Southern Hemisphere and has been recently reported in the Northern Hemisphere (NW Europe). In continental Chile, its first report dates back from 2000 for the locality of Antofagasta (23°S). Although there was no evidence about the vectors of introduction and spread, nor the source, some authors suggested maritime transport by ship hulls and aquaculture devices as putative introduction pathways and vectors. In the present study, we report for the first time the presence of *A. humilis* on the hull of an international ship in a commercial port in Concepción bay (36°S), south central Chile. We also found one individual associated to a seashell farm, 70 km far from Concepción bay. Further individuals were subsequently identified within Concepción bay: one juvenile settled upon international harbor pilings and a dozen individuals along aquaculture seashell longlines. For the first specimens sampled, species identification was ascertained using both morphological criteria and molecular barcoding, using the mitochondrial gene cytochrome c oxidase subunit I (COI) and a nuclear gene (ribosomal RNA 18S). The nuclear 18S gene and the mitochondrial gene COI clearly assigned the specimens to *A. humilis,* confirming our morphological identification. Two haplotypes were obtained with COI corresponding to haplotypes previously obtained with European and Northern Chilean specimens. The present study thus reports for the first time the presence of *A. humilis* in the Araucanian ecoregion, documenting the apparent expansion of this non-native tunicate in Chile over 2,000 km, spanning over three ecoregions. In addition we reveal the potential implication of the international maritime transport as a vector of spread of this species along the Eastern Pacific coast, and the putative role of aquaculture facilities in promoting local establishments of non-native tunicates.

## Introduction

The introduction of non-indigenous species has diverse and complex consequences on ecosystems and associated services (Simberloff et al., 2013; Katsanevakis et al., 2014). Once established, the containment and management of non-indigenous species in marine environments are particularly difficult and most often ineffective (Ojaveer et al., 2014). Consequently, management and control of the introduction vectors are of primary importance. In the marine realm, shipping and aquaculture trade are the pathways involved in most species introductions (Molnar et al., 2008; Occhipinti-Ambrogi & Galil, 2010; Nunes et al., 2014; Ojaveer et al., 2014). However, in most cases, the vectors (*sensu* Ojaveer et al., 2014) are difficult to ascertain with confidence. This is exemplified by non-native tunicates which are important members of the fouling community associated with artificial substrata and man-made structures both in ports or marinas and in shellfish farms (Carman et al., 2010; Shenkar & Swalla, 2011; Cordell, Levy & Toft, 2013). Being efficient fouling species, and despite limited natural dispersal ability, they are putatively easily spread, at both regional or global scales, through different pathways, like aquaculture trade (e.g., scallop farms in Chile, Clarke & Castilla, 2000) and shipping (Coutts & Dodgshun, 2007), and multiple vectors (e.g., ballast tank, sea chest, aquaculture equipment, stock exchanges etc.).

The ascidian *Asterocarpa humilis* (Heller, 1878), originally described in New Zealand (formerly as S*tyela humilis*), was later reported in different coastal regions around the world in SE Pacific (Chile), SE Atlantic (South Africa), and recently in NE Atlantic (France and Great-Britain) (Kott, 1985; Clarke & Castilla, 2000; Bishop et al., 2013; Turon et al., 2016). There were no direct observations of the species on vectors but it was reported (i) in its putative native range (New Zealand) on the hull of commercial ships as well as on aquaculture devices and (ii) in its introduction range both in marinas and on infrastructure associated with aquaculture: these observations suggest that both shipping and aquaculture trade could have been paths of entry (Clarke & Castilla, 2000; Bishop et al., 2013).

**Figure 1:**
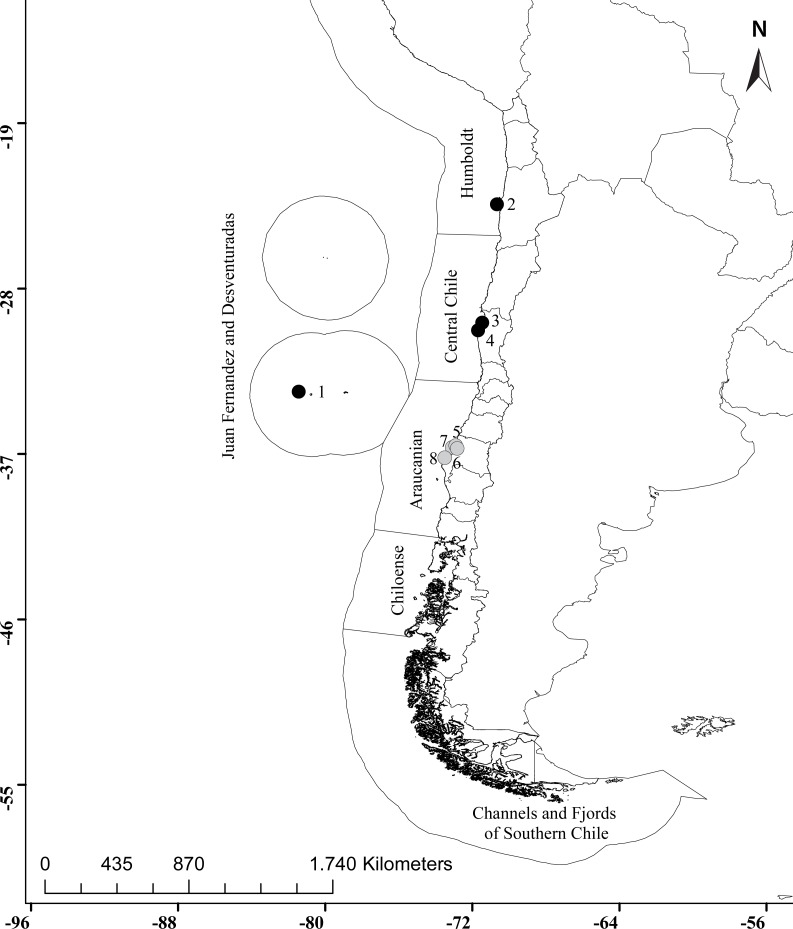
Distribution of *Asterocarpa humilis* along the Chilean coast. Black circles correspond to locations where *A. humilis* has been previously reported (1, Archipiélago Juan Fernández, Van Name, 1945; 2, Antofagasta, Clarke & Castilla, 2000; 3, Muelles UCN, Coquimbo; 4, Bahia Tongoy, Turon et al., 2016) and gray circles indicate the new four localities reported in this study (5, Coliumo; 6, Lirquén; 7, Talcahuano port; 8, Llico). Ecoregions are also shown (Spalding et al., 2007).

In Chile, *A. humilis* is one of the eight tunicate species reported so far as non-indigenous species (*Botryllus schlosseri*, *Ciona robusta* (formerly known as *C. intestinalis* type A), *Corella eumyota*, *Diplosoma listerianum*, *Lissoclinum perforatum*, *Molgula ficus*, *Pyura praeputialis* and *Asterocarpa humilis*) (Castilla & Neill, 2009; Bouchemousse, Bishop & Viard, 2016; Turon et al., 2016) along the coast. As shown in Fig. 1, this species had been reported in the Archipelago of Juan Fernández, located in the Juan Fernández and Desventuradas ecoregion, and in the northern localities of Antofagasta and Coquimbo in the Humboldtian ecoregion (Van Name, 1945; Clarke & Castilla, 2000; Turon et al., 2016). In Antofagasta (23°S) and Coquimbo (30°S) *A. humilis* was found attached to artificial substrates, on ropes in scallop farms (Clarke & Castilla, 2000; Turon et al., 2016). So far, there was no report of this species associated with vessels in Chile. In the present work, we provide the first report of *A. humilis* attached to the hull of an international commercial vessel docked in Talcahuano port in south central Chile (western Concepción bay, 36°S). In addition, we observed (a) an individual of this species in an aquaculture farm located in Llico, approximately 70 km southward Talcahuano port, (b) a juvenile on a settlement panel deployed along pilings within Lirquén port (eastern Concepción bay) and (c) an apparently well-established population (a dozen individuals) in another aquaculture farm in Coliumo (c.a. 20 km northward). This is thus also the first report of the establishment of *A. humilis* in the Araucanian ecoregion in Chile (Spalding et al., 2007).

## Materials & Methods

Between December 2014 and October 2015, surveys and samplings were done on the hulls of three international vessels arrived into Talcahuano port (36°43′S, 73°07′W). The shipping routes of these vessels were traced back and the type of antifouling used (paint or silicone) was also recorded (Table S1). Samples of the biofouling community were collected by scraping in the following areas of the hull: bilge keel, sea chest, propeller/rope guard and rudder.

For comparative purposes with the surveys made on the hulls, 18 settlement panels (10 cm × 10 cm) made of polypropylene were suspended at mid water depth in an area close to the sampling area of ships in Talcahuano bay, between April 16 and June 16, 2015. In addition, in 2016–2017, surveys and samplings of the subtidal hard bottom communities were made by scuba diving in seven localities along approximately 100 km of shoreline (Fig. S1): Talcahuano port, Coronel (37°1′49″S, 73°9′14″W), Chome (36°46′25″S, 73°12′49″W), San Vicente (36°45′33″S, 73°9′18″W), Lirquén (36°42′36″S, 72°58′57″W), Coliumo (36°32′16″S, 72°57′26″W) and El Manzo yacht club (a locality 1 km apart from the Talcahuano port). Finally, two aquaculture facilities (capture-based floating longlines) located nearby Llico (37°9′15″S 73°34′8″W), c.a. 70 km southward Talcahuano port, and nearby Coliumo (c.a. 20 km northward) were surveyed (Fig. S1).

During these surveys, ascidian specimens were collected in plastic bags and transported back to the laboratory of the Faculty of Sciences at the Universidad Católica de la Santísima Concepción for further species identification. The specimens were first characterized under binocular according to morphological traits, as described notably in the online resource 1 and 2 in Bishop et al. (2013) and by Turon et al. (2016), which provide comprehensive recent morphological descriptions and references.

A piece of branchial basket tissue was preserved in ethanol 95% for subsequent molecular analyses (performed on specimens collected prior 2017). We indeed ascertain the species identification by a molecular DNA barcoding approach following the method detailed in Bishop et al. (2013). Combining morphological and molecular data, these authors validated the use of COI to distinguish *A. humilis* and provided reference data for COI and 18S genes for this species. Briefly, total DNA was extracted using the “Nucleospin 96 Tissue core kit” (Macherey-Nagel, Düren, Germany) following the manufacturer’s protocol with a final elution in 100 µl. Two markers were used, the mitochondrial gene cytochrome c oxidase subunit I (COI) and the ribosomal RNA gene 18S. For the COI gene, PCR amplification was performed with the specific primer pair Ah-COIF (5′-CTAATTCGTACTGAGCTTTC-3′) and Ah-COIR (5′-GTTACTAATACCGTCCAACA-3′) developed by Bishop et al. (2013) and which produces a fragment of 467 base pairs (bp). For 18S, we used two primer pairs: 18S1 (5′-CCTGGTTGATCCTGCCAG-3′) and 18S4 (5′-GATTAAAGAAAACATTCTTGGC-3′) (Tsagkogeorga et al., 2009), and 18S-A (5′-CAGCAGCCGCGGTAATTCCAGCTC-3′) and 18S-B (5′-AAAGGGCAGGGACGTAATCAACG-3′) (Wada et al., 1992). The two overlapping fragments of the 18S gene were amplified over a total length of 1640 bp. PCR conditions for the two genes are described in the online resource 1 in Bishop et al. (2013). PCR products were visualized by electrophoresis in a 1.5% agarose gel. Direct Sanger sequencing was performed in both directions at Eurofins Genomics (Berlin, Germany).

Sequences were checked with the software CodonCodeAligner 5.1.4 (CodonCode Corporation, Dedham, MA, USA) and aligned using BioEdit (Hall, 1999). Finally, sequences were analyzed by BLAST in GenBank (https://blast.ncbi.nlm.nih.gov/Blast.cgi) which includes the sequences reported in Bishop et al. (2013) for European and New Zealand samples of *A. humilis*, as well as samples from northern Chile (Coquimbo; Turon et al., 2016). Note that we did not use the BOLD database (Barcoding of Life Database) as the two *A. humilis* sequences deposited in BOLD are those from Bishop et al. (2013), already obtained from GenBank.

Besides computing similarity indices, for displaying the results (i.e., similarities with previous published results) in a graphical way, we constructed a neighbor-joining tree of the Styelidae family, using MEGA v 6.06 (Kumar, Tamura & Nei, 2004). For the nuclear 18S gene, a 583 base-pair fragment was considered for tree reconstruction to allow comparison with a large number of sequences available in the GenBank dataset, including particularly the sequence for *Cnemidocarpa humilis* (actually *Asterocarpa humilis;* see Bishop et al., 2013) reported in Tsagkogeorga et al. (2009) (GenBank Accession No. FM244859) and the haplotype named Ah-H1 found in all *A. humilis* European samples examined by Bishop et al. (2013) (GenBank Accession No. JX312280.1). Similarly, a neighbor-joining tree was constructed for the COI fragment, considering 394 base pairs and including the two haplotypes previously identified on European samples by Bishop et al. (2013) (GenBank Accession No. JX312278.1, JX312279.1) and Turon et al. (2016) (GenBank Accession No. KU299758.1, KU299759.1). GenBank Accession numbers of the sequences used are indicated on Figs. S2 and S3.

## Results

Three tunicate specimens, all looking like *A. humilis*, were observed and collected alive during the ship surveys (2014–2015). Their size ranged from 2 cm to 3.5 cm. They all come from the sea chest of the hull of one oil tanker (Length: 206 m, Width: 29.69 m, Depth: 11.53 m; Oil tanker 1 in Table S1) docked in Talcahuano port, in Concepción Bay. The ship had a maritime route restricted to the eastern Pacific but connecting Chile and Canada, thus the Northern and Southern Hemispheres. Antifouling paint and silicone were the two antifouling methods used by the sampled ships. No specimens of *A. humilis* type were observed on the panels settled in the port in 2015 nor in hard bottom communities surveyed around in 2016. However, during this survey, one alive specimen of 1 cm was found attached on an aquaculture longline in the locality of Llico (Fig. S1). In 2017, one juvenile (<0.5 cm) was found in Lirquén port, facing Talcahuano port within Concepción Bay. In addition, a dozen adult specimens were identified along artificial substrata deployed for capture-based aquaculture purposes along a longline in Coliumo.

**Figure 2:**
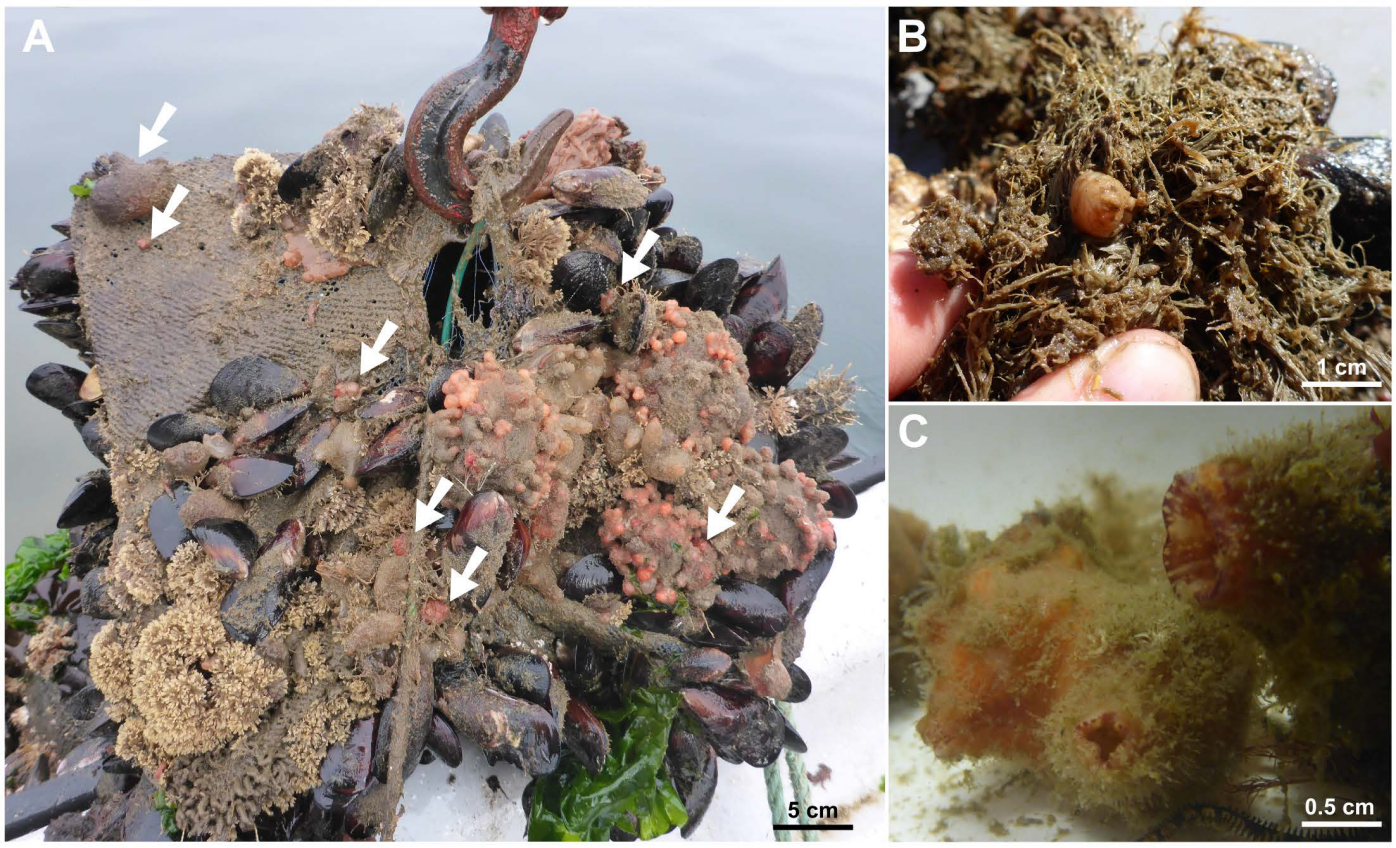
Specimens of *Asterocarpa humilis* collected in the field. Individuals collected upon artificial substrata in capture-based aquaculture farms in Coliumo (A, individuals indicated by arrows) and Llico (B). (C) Close-up of the siphons of relaxed individuals within sea water back to the laboratory.

Collected specimens displayed the morphological characteristics described for this species (according to Bishop et al., 2013; Turon et al., 2016). For instance, they display a firm tunic whitish on the ventral side and red-orange towards the siphons that was generally fouled by various epibionts (e.g., *Clytia linearis*, *Amathia* cf. *gracilis*, Fig. 2). The branchial basket was composed on each side by four branchial folds, each bearing about 9–11 longitudinal vessels and separated by ca. 2–3 longitudinal vessels. The dorsal lamina was simple and smooth-edged. On every specimen examined, the dorsal tubercle appeared U-shaped and its horns were oriented outwards. Gonads were present in clumps along the ventral midline on the right side of the body, as well as above the primary loop of the gut on the left side of the body. A few gonoducts (generally pinkish) could be seen oriented towards various directions. Conspicuous endocarps were present on the left side of the body adjacent to the gut, especially along the first half (descending arm) of the secondary loop. The anus had two thick whitish lips, outwardly curled, ever straight or curved. Also note that siphons display typical color patterns with wide primary and often fine secondary yellow-white stripes with a crimson background (this pigmentation generally persists on preserved specimens). This latter characteristic is one of the easiest to notice and discriminate *A. humilis* from other native species, thus is an interesting trait to examine in the field to warrant the observer to have a closer examination (Fig. 2).

In addition to morphological analyses, molecular DNA barcoding analyses were performed on the first specimens collected, i.e., on the three specimens collected on the ship hull and the individual found in Llico. Both COI and 18S gene sequences matched with 100% identity to *A. humilis* sequences recorded in GenBank. We found two different haplotypes of the COI gene analyzed, differing from each other by four nucleotides and identical to the GenBank Accession numbers JX312278 and KU299758. For the 18S gene, a single haplotype was obtained from the four study specimens. This haplotype is 100% identical (over 583 bp) to the *A. humilis* sequence available in GenBank (GenBank Accession no. JX312280.1). Results are displayed using neighbor-joining trees provided in Figs. S2 and S3, for COI and 18S respectively. Considering these results, and the perfect match between morphological and genetic identification of specimens, samples collected in 2017 were assigned to *A. humilis* based solely on their morphological characteristics.

## Discussion

We clearly identified as *Asterocarpa humilis* several specimens sampled along aquaculture longlines (Llico, Coliumo), three specimens collected on the hull of an international commercial ship docked in Talcahuano port, and a juvenile collected on a piling within Lirquén port. DNA sequences obtained from all DNA-barcoded specimens were identical with sequences available in GenBank. Interestingly, the two COI haplotypes found in our study were previously reported in Europe (GenBank Accession no. JX312278; Bishop et al., 2013) and northern Chile (Coquimbo; GenBank Accession no. KU299758; Turon et al., 2016) (Fig. S2). For the 18S gene, the single haplotype obtained from the four study specimens had been also previously described in both Europe (Bishop et al., 2013) and Chile (Turon et al., 2016) (Fig. S3). These two markers are however too poorly polymorphic at the species level to make any inferences regarding the introduction routes and sources based on these data. Because the species may be a selfer (Bishop et al., 2013), highly polymorphic markers, like microsatellites or Rad-seq markers, need to be developed to further examine the source of the introduction and the patterns of spread in Chile.

This study contributes to the increasing list of studies showing the benefit to use molecular barcoding for fast and effective identification of non-indigenous species (Comtet, Sandionigi & Viard, 2015). Most often, an important limitation for carrying out molecular barcoding is the availability of reference data. In our study, we could rely on a previous study that aimed to ascertain the accuracy of COI to distinguish *A. humilis* and which provides reference data (Bishop et al., 2013). Including molecular barcoding in biosecurity program, notably with the aim to make early-detection and prevention against non-indigenous species introduction, is increasingly recommended (Comtet, Sandionigi & Viard, 2015; Ojaveer et al., 2014; Lehtiniemi et al., 2015) but requires reliable molecular reference data. Researches aiming to develop such reference data, which needs support of taxonomic expertise, are however still rare (but see Dias et al., 2017 for an application in Western Australia).

Although *A. humilis* had been mainly reported from the north and north central Chile, this is the first report of its presence for the Araucanian ecoregion. The present findings extend the current distribution of *A. humilis* in Chile: it is now ranging over more than 2,000 km of coast from Antofagasta to Concepción (Llico), thus spreading over two biogeographic provinces and three ecoregions. The status of the species as an established species in Concepción area and its potential to locally expand and spread are however uncertain. On the one hand, we found a relatively dense population and we observed juveniles within a shellfish farm in Coliumo bay and in Lirquén port, thus indicating the presence of a reproductive population. On the other hand, only scattered individuals were encountered on other artificial substrata and the species remains virtually absent from neighboring natural habitats. As far as the present records can tell, this situation in Chile contrasts with other areas of introduction in Europe. For instance, in NE Atlantic, *A. humilis* is spreading fast at a regional level and establishes dense populations only a few years after its putative introduction in the surveyed area (Bishop et al., 2013). Thus, the low presence observed in our study could hardly be explained only by the age (i.e., recentness) of the introduction. This may suggest that *A. humilis* did not find, so far, suitable environmental conditions to establish as stable and dense populations in SE Pacific as in NE Atlantic. This hypothesis is difficult to ascertain and is, to some extent, questionable regarding that many invasive species, in particular tunicates, are shared by NE Atlantic and SE Pacific (e.g., *Corella eumyota*: Dupont et al., 2007, *Ciona robusta*: Bouchemousse, Bishop & Viard, 2016). Another hypothesis is that *A. humilis* is unable to successfully compete for space with native species commonly found at high density around the subtidal zone in Chile, such as native ascidians, barnacles and mussels (Castilla, 1999; Navarrete & Castilla, 2003; Valdivia et al., 2005; Caro et al., 2008). From the current knowledge, only a few species have been found to successfully spread along SE Pacific coasts, and rare are the reports about abundant or ‘pest’ species (e.g., the green alga *Codium fragile* spp. *tomentosoides* in *Gracilaria chilensis* aquaculture facilities reported by Castilla & Neill (2009) or the tunicate *Ciona robusta* observed by Dumont, Gaymer & Thiel (2011) along scallop cultivation installations). As a well-studied example, the distribution of the tunicate *Pyura praeputialis*, introduced from Australia a few centuries ago, is noteworthy: this species is restricted to mid-low intertidal of the Bay of Antofagasta (Castilla et al., 2004; Caro et al., 2011). Given the records hereby made upon novel substrata deployed for seashell capturing purpose within aquaculture farms, competition experiments could be particularly interesting to carry out to examine different hypotheses regarding the invasion potential of *A. humilis* in SE Pacific. Since *A. humilis* seems to successfully settle and grow on the tunic of the native ascidians *Pyura chilensis* (Fig. 2A, see also Turon et al., 2016), facilitation processes should also be taken into account in such studies (Bulleri, Bruno & Benedetti-Cecchi, 2008).

Biofouling on ships’ hulls has been recognized to have large potential for spreading non-indigenous species and promote invasion (Minchin & Gollasch, 2003). Our study is the first evidence that the hull of commercial vessels could be an effective vector of introduction and spread of *A. humilis* in SE Pacific coasts. In this context, it is noteworthy that the vessel on which the *A. humilis* specimens were found is connecting Canada to Chile and vice versa. To our knowledge, *A. humilis* has never been reported so far in Canada. It is thus unlikely that the source of the individuals sampled in the sea-chest in Concepción originates from Canada. It is also unlikely that these individuals had been seeded by local harbor populations, as our surveys failed to find any, except one juvenile in another port. Also no individuals were observed on the experimental panels. So if the species is present in the port, it is likely rare. Considering that *A. humilis* broods its young in the atrial cavity, and thus is likely a very short disperser, it is unlikely that the individuals found in the sea-chest came from reproduction of a local (port) population. As a most likely alternative hypothesis, these individuals might have been produced by individuals located in Antofagasta bay. *A. humilis* is indeed established for decades (Clarke & Castilla, 2000), and thus possibly at higher density, in this bay which is located along the regular maritime route of the surveyed ship.

Invasions by sea squirts are repeatedly associated with aquaculture activity and maritime trade (Carlton & Geller, 1993; Lambert & Lambert, 1998; Coutts & Dodgshun, 2007) and, concerning *A. humilis,* the roles of ship hulls and commercial ports have already been suggested (Clarke & Castilla, 2000). Our results demonstrate the importance of international shipping as putative vector of introduction for *A. humilis* in the Eastern Pacific. It is noteworthy that we found this species in sea-chest only. Previous studies have also reported non-indigenous species present only in sea-chest, for instance, the clam *Corbula gibba* and the green crab *Carcinus maenas*, both native to Europe, were found only in sea-chests during a survey carried out in Australia (Coutts, Moore & Hewitt, 2003). The same was reported by Sylvester et al. (2011) with one specimen of the mollusk *Rapana venosa* found in a sea-chest on a ship surveyed in the Vancouver port. Sea-chests represent unique micro-habitats on vessels as anti-fouling paints are there poorly efficient owing to specific water-flows (Coutts & Dodgshun, 2007). Continuously supplied by food and oxygen, and protected from strong water flows, diverse fouling organisms may find favorable conditions to develop self-sustainably in these micro-habitats. Whilst the origin of the specimens found on the study ship hull remains elusive on the basis of the present report, we warrant further consideration regarding the role of sea-chest in invader transport over long distances.

## Conclusions

There are few records of non-native marine species in the SE Pacific coast as compared to other regions of the world. In addition, direct evidences of the main transport vectors promoting novel introductions are lacking. In this work, we report and confirm by genetic analyses the presence and spread of the ascidian invader *A. humilis* in Chile along more than 2,000 km of coast. We also found evidence to suggest that international maritime transport activity could be one of the vectors influencing species introduction in Chile, and more specifically *A. humilis*. Although this species may reach great abundances in other introduced areas (e.g., Europe), it has not—as far as the current information can tell—established important populations in Chile. Native biotic resistance or low environmental similarity between source and donor regions are still hypotheses to be tested to explain invasion patterns in the SE Pacific.

##  Supplemental Information

10.7717/peerj.3672/supp-1 Figure S1 Survey sites (harbors, aquaculture facilities and natural habitats) Gray circles correspond to locations where *A. humilis* was encountered (1 = Coliumo, 2 = Lirquén port, 3 = Talcahuano port, 7 = Llico) and black circles indicate visited sites where *A. humilis* is absent (4 = San Vicente port, 5 = Chome, 6 = Coronel port).

10.7717/peerj.3672/supp-2 Figure S2 Phylogenetic tree of cytochrome c oxidase subunit I (COI) haplotypes for the family Styelidae, constructed by the neighbor-joining method Sequences obtained in the present study are identified in the tree as Ah COI ShipCH (1 to 3) and FarmCH, and Accession Numbers refer to sequences from GenBank. Two haplotypes were found: one (Ah COI ShipCH3) is identical to the H1 haplotype obtained from European specimens (JX312278; Bishop et al., 2013) and the other one, found in three study specimens, is identical to one haplotype from Northern Chile (KU299758; Turon et al., 2016).

10.7717/peerj.3672/supp-3 Figure S3 Phylogenetic tree of 18S haplotypes for the Styelidae family, constructed by the neighbor-joining method Sequences obtained in the present study are identified in the tree as Ah COI ShipCH (1 to 3) and FarmCH, and Accession Numbers are indicated for other sequences mined from GenBank. Note that all the sequences obtained in our study are identical among them and with the sequence Ah-H1 18S JX312280.1 (obtained using European specimens by Bishop et al., 2013 and using Chilean specimens from Coquimbo by Turonetal2016).

10.7717/peerj.3672/supp-4 Table S1 Characteristics of surveyed ships and hulls Sampling dates, information on the last arrivals (port, country and date, when available) and the type of antifouling used by each surveyed ship (silicone or paint) are indicated.

## Additional Information and Declarations

### Data Availability

The following information was supplied regarding data availability: DNA sequences obtained were identical to sequences already available in GenBank.
