# Supplementary figures and images for: Presence of the tunicate Asterocarpa humilis on ship hulls and aquaculture facilities in the coast of the Biobío Region, south central Chile
Source: PeerJ. 2017 Aug 14;5:e3672. doi: 10.7717/peerj.3672 (PMC5560234; doi:10.7717/peerj.3672)

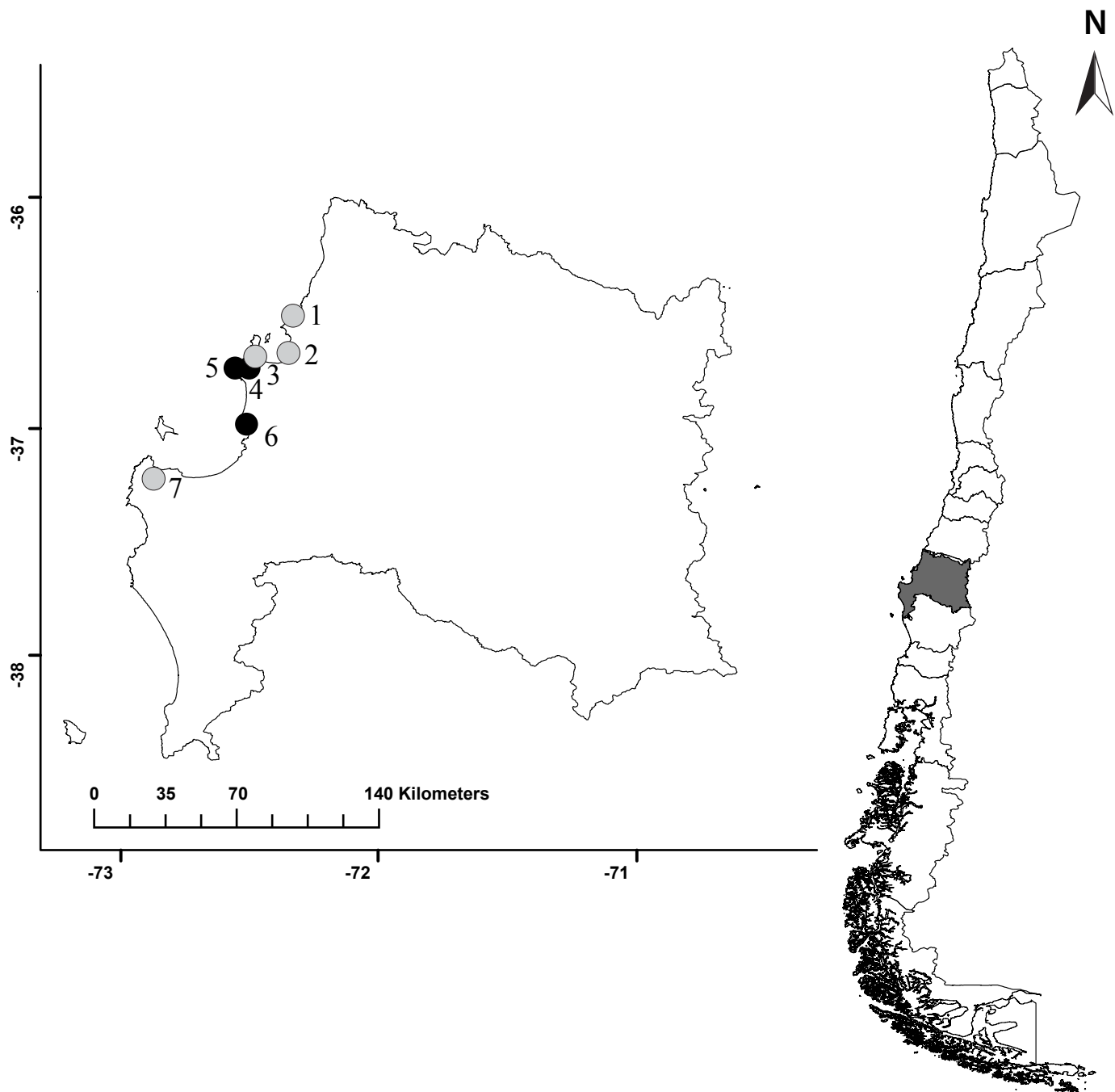

Supplement: Figure S1 — Gray circles correspond to locations where A. humilis was encountered (1 = Coliumo, 2 = Lirquén port, 3 = Talcahuano port, 7 = Llico) and black circles indicate visited sites where A. humilis is absent (4 = San Vicente port, 5 = Chome, 6 = Coronel port). [file peerj-05-3672-s001.pdf]

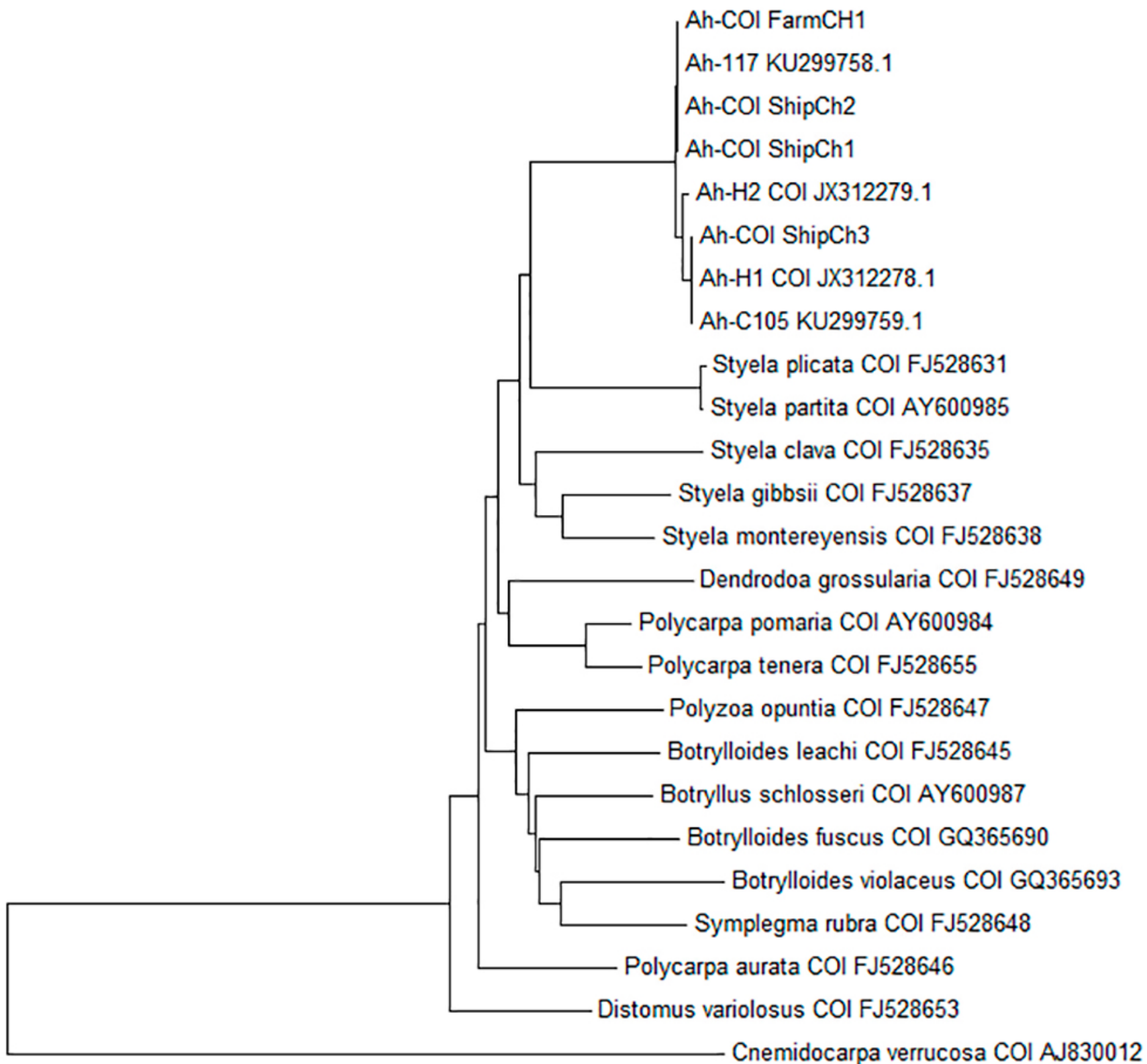

0.10

Supplement: Figure S2 — Sequences obtained in the present study are identified in the tree as Ah COI ShipCH (1 to 3) and FarmCH, and Accession Numbers refer to sequences from GenBank. Two haplotypes were found: one (Ah COI ShipCH3) is identical to the H1 haplotype obtained from European specimens (JX312278; Bishop et al., 2013) and the other one, found in three study specimens, is identical to one haplotype from Northern Chile (KU299758; Turon et al., 2016). [file peerj-05-3672-s002.pdf]

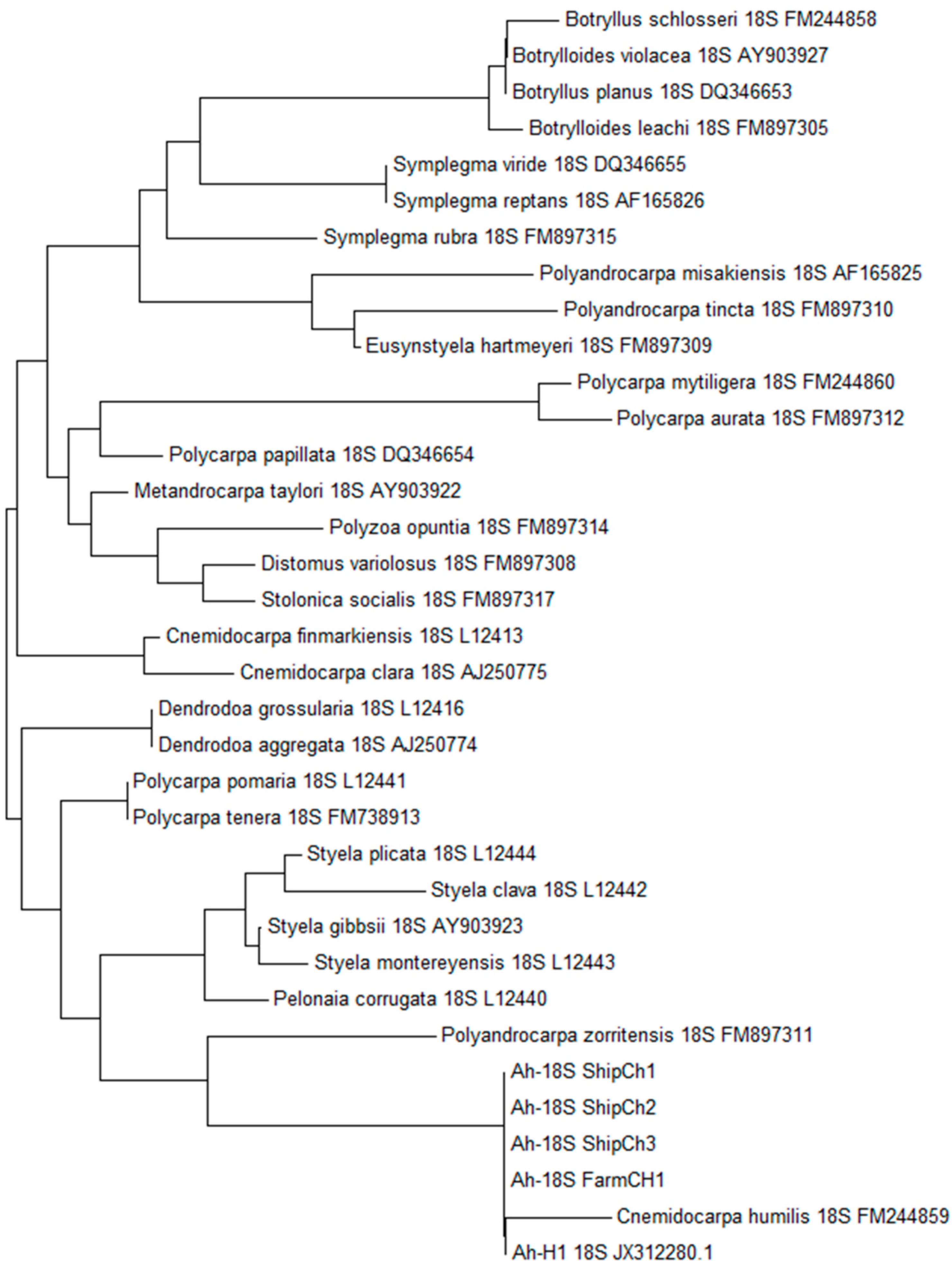

0.0020

Supplement: Figure S3 — Sequences obtained in the present study are identified in the tree as Ah COI ShipCH (1 to 3) and FarmCH, and Accession Numbers are indicated for other sequences mined from GenBank. Note that all the sequences obtained in our study are identical among them and with the sequence Ah-H1 18S JX312280.1 (obtained using European specimens by Bishop et al., 2013 and using Chilean specimens from Coquimbo by Turonetal2016). [file peerj-05-3672-s003.pdf]
