# Supplementary material for: Presence of the tunicate Asterocarpa humilis on ship hulls and aquaculture facilities in the coast of the Biobío Region, south central Chile
Source: PeerJ. 2017 Aug 14;5:e3672. doi: 10.7717/peerj.3672 (PMC5560234; doi:10.7717/peerj.3672)
Supplement: Table S1 — Sampling dates, information on the last arrivals (port, country and date, when available) and the type of antifouling used by each surveyed ship (silicone or paint) are indicated. [file peerj-05-3672-s004.docx]

| **Ship** | **Sampling date** | **Last ports** | **Country** | **Date of arrival** | **Antifouling type** |
| --- | --- | --- | --- | --- | --- |
| Frigate | 13-12-2014 | Valparaíso | Chile | 08-04-2014 | Paint |
|  |  | Callao | Peru | 24-04-2014 |  |
|  |  | Manta | Equator | 03-05-2014 |  |
|  |  | Cartagena de Indias | Colombia | 15-05-2014 |  |
|  |  | La Guaira | Venezuela | 24-05-2014 |  |
|  |  | Santo Domingo | Dominican Republic | 02-06-2014 |  |
|  |  | Veracruz | Mexico | 18-06-2014 |  |
|  |  | Colon | Panama | 03-07-2014 |  |
|  |  | Cruce Canal | Panama | 04-07-2014 |  |
|  |  | Balboa | Panama | 05-07-2014 |  |
|  |  | Guayaquil | Equator | 16-07-2014 |  |
|  |  | Cumberland | Chile | 04-08-2014 |  |
|  |  | Talcahuano port | Chile | 13-12-2014 |  |
| Oil tanker 1 | 13-12-2014 | Antofagasta | Chile | NA | Paint |
|  |  | Callao | Peru | NA |  |
|  |  | Cartagena de Indias | Colombia | NA |  |
|  |  | Long Beach | USA | NA |  |
|  |  | NA | Canada | NA |  |
|  |  | Talcahuano port | Chile | NA |  |
| Oil tanker 2 | 02-05-2015 | Bahía Blanca | Argentina | NA | Silicone |
|  |  | Caleta Olivia | Argentina | NA |  |
|  |  | Talcahuano port | Chile | NA |  |
